# The Address of Drs. Shattuck and Lipscomb

**Published:** 1873-09

**Authors:** 


*The Address of Drs. Lipscomb and Shattuck.*

We call attention to the chaste, erudite and noble address of our distinguished Mississippi friends, delivered before that splendid body of medical gentlemen, the Lowndes County Medical Society. We feel sure our readers, everywhere, will deem this address, alone, worth the subscription price of the RECORD; and we ask them to place it in the hands of their medical brethren, that its noble sentiments and pure lessons of medical ethics may have their "perfect work" in all hearts and in all sections of our country.

